# Semantic segmentation of reflectance confocal microscopy mosaics of pigmented lesions using weak labels

**DOI:** 10.1038/s41598-021-82969-9

**Published:** 2021-02-11

**Authors:** Marissa D’Alonzo, Alican Bozkurt, Christi Alessi-Fox, Melissa Gill, Dana H. Brooks, Milind Rajadhyaksha, Kivanc Kose, Jennifer G. Dy

**Affiliations:** 1 Draper Laboratory, Cambridge, MA 02139 USA; 2 Electrical and Computer Engineering, Northeastern University, Boston, MA 02115 USA; 3 Paige AI, New York City, NY 10036 USA; 4 Memorial Sloan Kettering Cancer Center, New York City, NY 10022 USA; 5 Caliber I.D. Inc., Rochester, NY 14623 USA; 6 Department of Pathology at SUNY Downstate Medical Center, New York, 11203 NY USA; 7 Skin Medical Research Diagnostics, P.L.L.C., Dobbs Ferry, 10522 NY USA; 8 Faculty of Medicine and Health Sciences, University of Alcala, Madrid, Spain

**Keywords:** Cancer imaging, Computational science

## Abstract

Reflectance confocal microscopy (RCM) is a non-invasive imaging tool that reduces the need for invasive histopathology for skin cancer diagnoses by providing high-resolution mosaics showing the architectural patterns of skin, which are used to identify malignancies in-vivo. RCM mosaics are similar to dermatopathology sections, both requiring extensive training to interpret. However, these modalities differ in orientation, as RCM mosaics are horizontal (parallel to the skin surface) while histopathology sections are vertical, and contrast mechanism, RCM with a single (reflectance) mechanism resulting in grayscale images and histopathology with multi-factor color-stained contrast. Image analysis and machine learning methods can potentially provide a diagnostic aid to clinicians to interpret RCM mosaics, eventually helping to ease the adoption and more efficiently utilizing RCM in routine clinical practice. However standard supervised machine learning may require a prohibitive volume of hand-labeled training data. In this paper, we present a weakly supervised machine learning model to perform semantic segmentation of architectural patterns encountered in RCM mosaics. Unlike more widely used fully supervised segmentation models that require pixel-level annotations, which are very labor-demanding and error-prone to obtain, here we focus on training models using only patch-level labels (e.g. a single field of view within an entire mosaic). We segment RCM mosaics into “benign” and “aspecific (nonspecific)” regions, where aspecific regions represent the loss of regular architecture due to injury and/or inflammation, pre-malignancy, or malignancy. We adopt Efficientnet, a deep neural network (DNN) proven to accurately accomplish classification tasks, to generate class activation maps, and use a Gaussian weighting kernel to stitch smaller images back into larger fields of view. The trained DNN achieved an average area under the curve of 0.969, and Dice coefficient of 0.778 showing the feasibility of spatial localization of aspecific regions in RCM images, and making the diagnostics decision model more interpretable to the clinicians.

## Introduction

Skin cancer is the most common cancer type in the world, with 4.6 million cases diagnosed worldwide each year, and 3.6 million of the cases reported in the United States^1^. The current “gold standard” for diagnosing these cancers is biopsy followed by histopathology, in which the tissue is physically excised from the patient, followed by histopathologic processing and interpretation. This process is highly accurate, but the procedure is invasive, scarring, and stress-inducing for the patient. Additionally, the benign-to-malignant biopsy ratio is extremely variable, ranging from as low as 2-to-1 to as high as 47-to-1 in adults and even 600-to-1 in children depending on the clinician’s experience, as well as lesion type and the characteristics of the patient^2^. Therefore, in clinical practice, there is a need for in vivo imaging methods that could help reduce the number of benign lesions referred to biopsy.

Among several in-vivo optical microscopy methods, reflectance confocal microscopy (RCM) is being adopted as a clinical tool especially promising for this task because it provides resolution and sectioning comparable to that of histopathology. Similar to histopathology, RCM imaging-based diagnoses are based on the architectural patterns of cell clusters in the skin and the morphological appearance of the individual cells within those patterns. However, while histopathology images are colored due to differential contrast provided by cellular cytoplasmic-, cell type- and nuclear- specific staining agents, RCM mosaics are single-channel (backscattered, visualized in grayscale) with the difference between refractive properties of the cellular cytoplasmic components as the only source of contrast. This limited differential contrast combined with the horizontal orientation introduces a learning curve for visual interpretation of RCM images. An investment in training is required to reach proficiency in reading these images and achieve high rates of accuracy as the experts. However, the length of time to reach proficiency or expertise depends on the background of the individual (e.g. dermatopathologist vs. non-dermatopathologist) and the time dedicated to reviewing RCM cases and attending training programs or fellowships. Non-dermatopathologists tend to have a steep learning curve compared to their counterparts. Thus a current challenge in the wider clinical use of RCM is to catalyze the adoption of the technology by this larger cohort of novice users^3^. Image processing and machine learning-based algorithms can ease this adoption by providing quantitative analysis tools to help the clinicians in tasks such as; analyzing RCM images for quality assurance^4,5^, segmenting architectural patterns in RCM images^3,6,7^, providing a swift and quantitative diagnostic analysis^6^ or secondary review.

Towards this end, in this paper, we investigate the feasibility and accuracy of implementing a CAM based weakly supervised semantic segmentation on RCM mosaics (note that a mosaic typically covers up to $64 \,{\text{ mm }}^{2}$ and contains on the order of 256 million pixels) collected at the dermal-epidermal junction (DEJ) to separate non-worrisome (“benign”) areas from those concerning for melanoma (“aspecific”). The goal is to associate each pixel in the image with one of the labels (benign vs aspecific). Machine learning based semantic segmentation methods can aid in the adoption of RCM imaging by not only classifying large mosaic images but, more helpfully, segmenting them into different architectural patterns, as lesions typically are comprised of multiple patterns of variable architecture. Automatically classifying each pixel in mosaics as benign or aspecific can help with both diagnosis and training by (1) enabling the clinician to rapidly identify the areas of most concern, (2) ensuring areas that could represent malignancy are not overlooked (improving sensitivity), and (3) helping novice clinicians by aiding them in their training. Automated semantic segmentation of RCM mosaics into different cellular morphological patterns that lead to diagnosis could further accelerate this process by adding much more transparency and interpretability to the process^3^.

Traditionally, semantic segmentation models are trained using a paired set of images and their pixel-wise annotation mask. Here we refer to this approach as the fully supervised training setting. However, obtaining pixel-wise image labels is challenging, especially for medical imaging applications. This issue is compounded in our application by the large size of our RCM mosaics (i.e., up to 12K-by-12K pixels). Moreover, the cellular morphological patterns of skin do not adhere to strict borders, with ill-defined transitions rather than hard borders, and identification of the patterns requires expert knowledge in reading these images. Therefore, obtaining pixel-wise annotation masks is a laborious and an error-prone process requiring extensive time from expert clinicians. This constitutes an imposing bottleneck to obtaining large supervised datasets for training semantic segmentation models that generalize well. Thus fully supervised semantic segmentation methods tend to be less adequately trained, less generalizable, and less able to adapt to the recognition of a different disease or a task on a different tissue type than simple classification networks.

**Figure 1:**
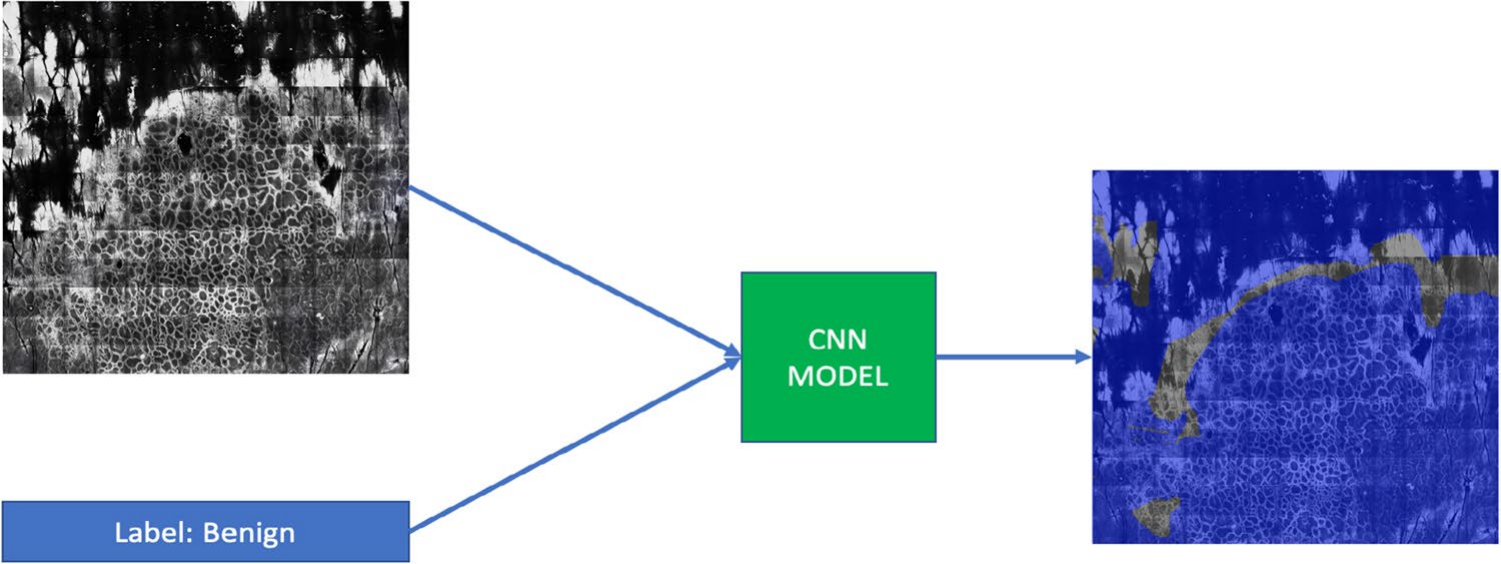
A visual description of weakly supervised learning—a convolutional neural network (CNN) model receives the image and an image-level label as input, and produces as an output pixel-level annotations, represented by the blue here. Regions that were not labeled by the expert readers are not colored. The block diagram is prepared using Powerpoint 16.44 (https://insider.office.com/en-us/releasenotes/mac/slow/version-16-44-build-20120602).

**Figure 2:**
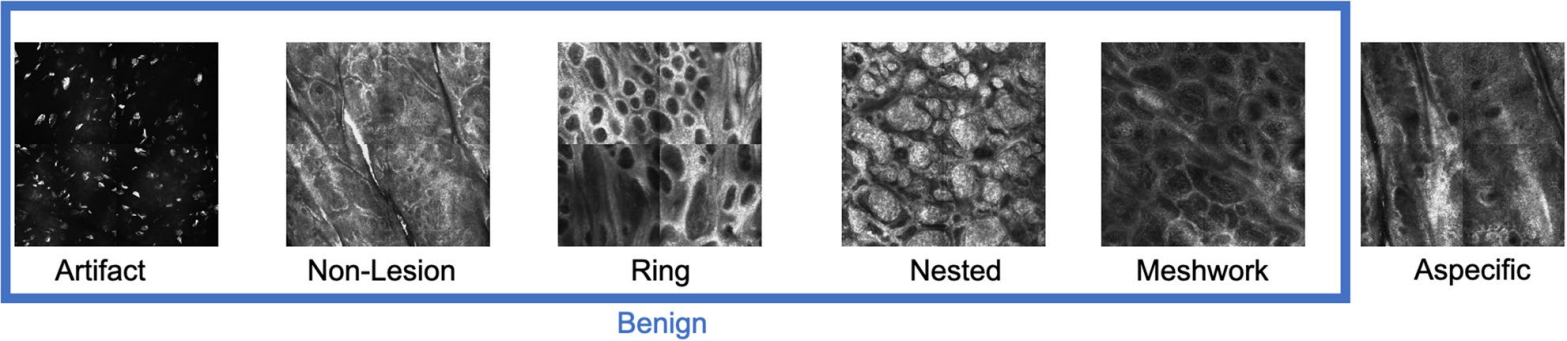
Six cellular morphological patterns classified by clinicians as viewed under reflectance confocal microscopy. For this research, the five leftmost patterns were grouped together into “benign” while “aspecific”, a precursor to malignancy, is the second class.

Weakly supervised learning (WSL) based semantic segmentation has been proposed as a promising alternative to reduce the labeling burden for clinicians while maintaining the interpretability of semantic segmentation^3^. As seen in Fig. 1, WSL models can be trained using data with only image-level (e.g. the whole mosaic or individual single fields of view in mosaic) labels in order to classify an image, while yielding pixel-wise label scores and localizing the corresponding regions of interest within the images. The main drawback of WSL is that it typically requires a more extensive volume of training data, in exchange for far fewer labels on that data, as it tries to extrapolate pixel-level segmentation from only image-level labels. In addition, the nature of RCM mosaics poses some unique challenges to training models. Most pathological or medical images, such as those of cells or organs, have known shapes with clear boundaries and distinct contrast between the region of interest and the background. Moreover, for organ segmentation, the spatial location of the target organ is approximately known within the field of view. In contrast, RCM images contain cellular patterns with complex shapes of various sizes with ambiguous boundaries that can change appearance under speckle noise and can appear at arbitrary spatial locations in the image^3^. Some examples of RCM images are shown in Fig. 2 to illustrate these challenges.

**Figure 3:**
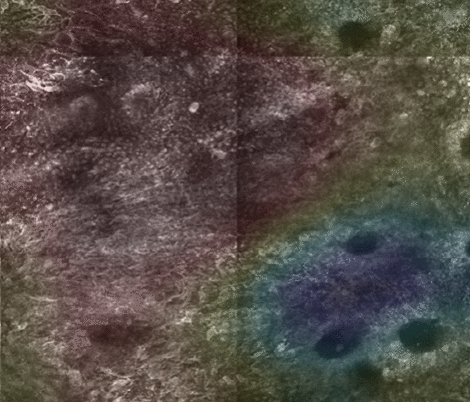
An example CAM for benign vs. aspecific classification. The CAM result is superimposed as a transparent color heatmap on the underlying greyscale RCM image. The model’s decision is aspecific, and the CAM highlights the contribution of each pixel to that decision. The blue-to-red color in the heatmap corresponds to low-to-high contribution (for the aspecific label), so the red areas correspond to where the aspecific regions are located.

One method for achieving weakly supervised semantic segmentation is by generating class activation maps (CAMs). When a convolutional neural network (CNN) is used to classify an image (e.g., benign vs. aspecific), CAMs visualize how much each pixel of that image contributes to the CNN’s classification decision. In doing so, a CAM approximates how suspicious each pixel is, thus performing a rough semantic segmentation. An example CAM for benign vs aspecific classification on an RCM image is given in Fig. 3. The model’s decision on the given image is aspecific, and the corresponding CAM highlights the contribution of each pixel to this final decision. The blue-to-red color in the heatmap means low-to-high contribution, where the red areas correspond to where the aspecific regions are located.

In this paper, we are interested in RCM mosaics collected at the DEJ level, which constitutes the boundary between the epidermis (epidermal anatomic layer) and the underlying papillary dermis (superficial stromal anatomic layer). DEJ is the most diagnostically important skin layer since most skin cancers, including melanoma, arise at the DEJ. Melanocytic lesions can harbor any one or a mixture of four previously defined patterns^8^ at the DEJ: ring, meshwork, nested (also referred to as clod), and aspecific (also referred to as nonspecific). Regular and architecturally preserved versions of ring, meshwork, and nested patterns are seen mostly in benign areas. However, these patterns can also show varying degrees of atypia or disruption, with increasing atypia and/or disruption making a malignant diagnosis more likely. The aspecific pattern is most frequently seen in melanoma, often result of complete disruption of the ring, meshwork, or nested patterns. However, the aspecific pattern can also be related to the anatomical site (e.g. face especially with severe sun damage), trauma, or inflammation in the tissue. Thus, areas that fall under these criteria were assigned to “aspecific” label. In order to segregate out benign areas, only ring, meshwork, and nested patterns that were free of atypia and disruption (i.e. architecturally preserved) were assigned to the *“benign”* label along with non-lesion and artifact areas (Fig. 2). Areas with mild atypia or disruption that did not fulfill the criteria for aspecific pattern were ignored. Detection and segmentation of aspecific patterns is a compelling goal because, in the absence of a history of trauma, they are more likely to correspond to regions containing malignant features. However, as the aspecific pattern is the most variable in appearance among all the morphological patterns at the DEJ, it can be difficult for clinicians to identify. Therefore, it is imperative that these areas are carefully scrutinized and never overlooked. Previously^3,4,6^, authors have successfully trained a fully supervised segmentation algorithm for identifying five benign patterns of interest and the aspecific pattern in RCM mosaics, image-level classification models to assess the RCM mosaic quality, and lesion level diagnosis algorithm for RCM mosaics, respectively. However, to our knowledge, this work is the first to attempt to achieve weakly supervised semantic segmentation on RCM images.

The rest of the paper is organized as follows: first, we briefly present the experiment design and the dataset, followed by the results. Then, we discuss the materials and methods used for training, including the algorithm, network architecture, and post-processing. and finally, we draw some conclusions and discuss future work.

## Results

We report on two sets of experiments. First, we assessed the image(patch)-level classification performance of the trained model. In this experiment, a patch is a small section of a mosaic consisting of 250,000 pixels. We fed the model with patches extracted from RCM mosaics and retrieved a single binary result (benign vs aspecific) per patch. The performance of the model is assessed in terms of patch-wise receiver-operating characteristic (ROC) curves as well as the area under the ROC curve (AUC). Following this initial analysis, we proceed with mosaic segmentation experiments where we process the mosaics in a sliding window (patch) fashion while generating CAMs for each patch fed into the model. These patch CAMs are then compiled into the final output mosaic to segment out the aspecific pattern regions in the mosaics. We provide experimental results for the CAM generation method for varying step sizes and levels of Gaussian smoothing parameters as defined in detail in “Materials and methods”. Besides the conventional Dice coefficient^9^ metric for segmentation, we also provide pixelwise ROC and AUC metrics since the output of the CAMs is a probability value rather than a binary segmentation value.

As detailed in the “Materials and methods” section we adopted an existing CNN called Efficientnet from the literature. The code for Efficientnet was adopted from^10^. All computation was performed on the cluster nodes equipped with Tesla K40 and workstations equipped with TITAN V and RTX6000 GPUs.

### Dataset

The data used in these experiments is composed of 157 RCM mosaics, obtained from 157 different patients. The data was collected under an IRB-approved clinical-study at Memorial Sloan Kettering Cancer Center (New York, NY, and Hauppauge, NY), the University of Rochester (Rochester, NY), Loma Linda University Health (Loma Linda, CA), and Skin Cancer Associates (Plantation, FL), with additional clinical cases contributed by the University of Modena and Reggio Emilia in Italy. Each mosaic thus comes from a unique skin lesion. These mosaics were partially annotated at a pixel-wise level by two expert readers for another study^3^ using the open-source software package Seg3D^11^. These mosaics, of varying sizes from $7000 \times 8000$ to 12,000$\,\times \,$12,000 pixels (14–$36\,{\text {mm}}^{2}$, respectively), were chosen to reflect the general diversity of data seen in typical clinical practice. The data collected in the USA was drawn from a larger dataset that includes cases of several different conditions and among those, we selected the benign and the melanoma cases to include in the study. The dataset from Italy contained similar clinical cases, and we randomly selected cases with a benign or melanoma diagnosis from this dataset. The breakdown of the labels among the classes in the original annotation can be found in Table 1.

We first partitioned the data patient-wise into training, validation, and test sets with 70%, 10%, and 20% of the data, respectively, in each category. We tried to preserve the ratio of pixel labels in each class in each set to remain similar to the overall data set to avoid any bias. Next, we downsampled the mosaics by a factor of 4 in each direction and converted our pixel-wise labeled data set to a weakly labeled one by selecting a random pixel with the desired label and collecting a patch of 500-by-500 pixel window (1 mm-by-1 mm in the downsampled mosaics) centered around the selected pixel. We labeled the patch with the label of the center pixel. Each patch was thus weakly labeled with a global label and no further pixel-wise labeling. Each patch may contain both aspecific and benign areas, so our segmentation aimed to increase precision by pinpointing the location of the aspecific areas within the whole field of view.

Following the generation of the weakly supervised data, the pixel-level labels were not used during training. However, they were used for evaluating accuracy. We created a balanced data set of these patches by extracting $\sim 5000$ such patches from each of the five types of benign labels (24,722 benign patches) and another 23,153 patches for the aspecific label. This resulted in 47,875 patches in the final data set. While extracting these patches, each mosaic contributed to the overall data set with a ratio directly proportional to its pixel-wise label content across six labels. In this way, we keep the balance of labels in the training, validation, and test sets similar to the overall dataset.

At each iteration of the training, we also conducted data augmentation by randomly cropping 256-by-256 pixel-wise areas within these initial larger patches to simulate the acquisition of single RCM images (field of view $\sim 0.5\,{\hbox {mm}}$-by-0.5 mm) as inputs to the segmentation model. The patch size and the downsampling ratio were carefully selected according to our previous experience with clinicians.

**Table 1:** Class distribution statistics: the top section of the table reports the distribution of labels. In the bottom section, we report the overall fraction of pixels that were labeled as well as the number of mosaics that were used in the study.

Pattern	Percentage (pixels)	Patients
Background	14% (71.7M)	106
Artifact	17 % (88.6M)	52
Mesh	17% (88.6M)	70
Nest	5% (23.9M)	70
Ring	34% (172.3M)	82
Benign (sum of above)	88% (445.1)	77
Aspecific	12% (59.9M)	80
Total Labeled	38% (505M)	157

Each mosaic was collected from a different patient.

### Patch-level classification performance

**Figure 4:**
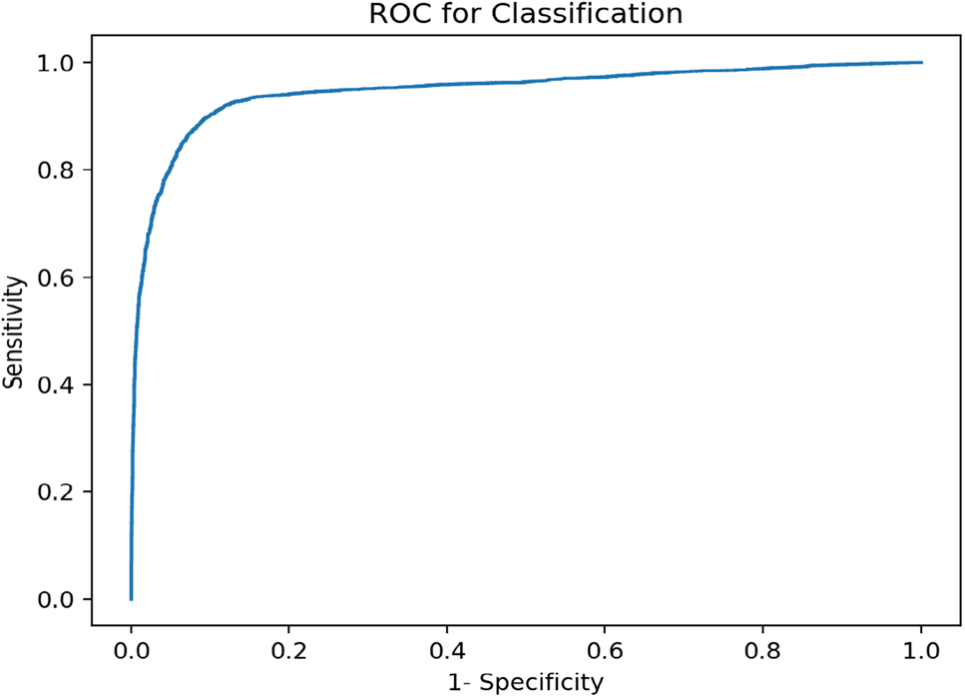
The ROC on the test set for the classification problem.

The patch-level classification on the test set mosaics achieved an accuracy of 88.44% with a softmax threshold of 0.5, meaning that if a patch received a score greater than 0.5, it was classified as “aspecific”, while a patch with a lower score was classified as “benign”. An ROC curve, shown in Fig. 4 was be created by varying this softmax threshold and the resulting AUC was 0.947, close to the ideal value of 1.

### Semantic segmentation performance

Segmentation maps on the test mosaics were generated in eighteen different trial runs over the test set, for combinations of different (1) CAM generation methods, (2) step size between patches, and (3) $\sigma$ used in Gaussian weighting. Two CAM generation methods were examined—the traditional CAM generation method of extracting the final feature extraction layer, and a more novel method called ScoreCAM. These methods are discussed in more detail in “Traditional class activation map generation” and “ScoreCAM generation”. Gaussian weighting was used due to its potential to reduce image noise. Step sizes of 25, 50 and 100 pixels were selected for testing based on results reported in our previous studies^7^ and^3^. The $\sigma$ values were chosen by visually inspecting the output of the CAMs—any values of $\sigma$ greater than ten consistently resulted in unrealistic blurring in the segmentation results. Table 2 shows the AUC and Dice results for all trials using the test mosaics. Rows without a $\sigma$ value (no Gaussian) indicate that averaging without Gaussian weighting was used for accumulation at the overlap areas. When evaluated at the patch-level for classification accuracy, the model correctly classifies 88.44% of patches.The Dice score was calculated for all thresholds between 0 and 1 in increments of 0.02, and the optimal score was selected. For the traditional CAM generation, this threshold was roughly 0.5, while the optimal threshold for ScoreCAM varied between 0.25 and 0.4. We also computed ROC curves in order to study pixel-wise sensitivity and specificity at different threshold levels.

**Figure 5:**
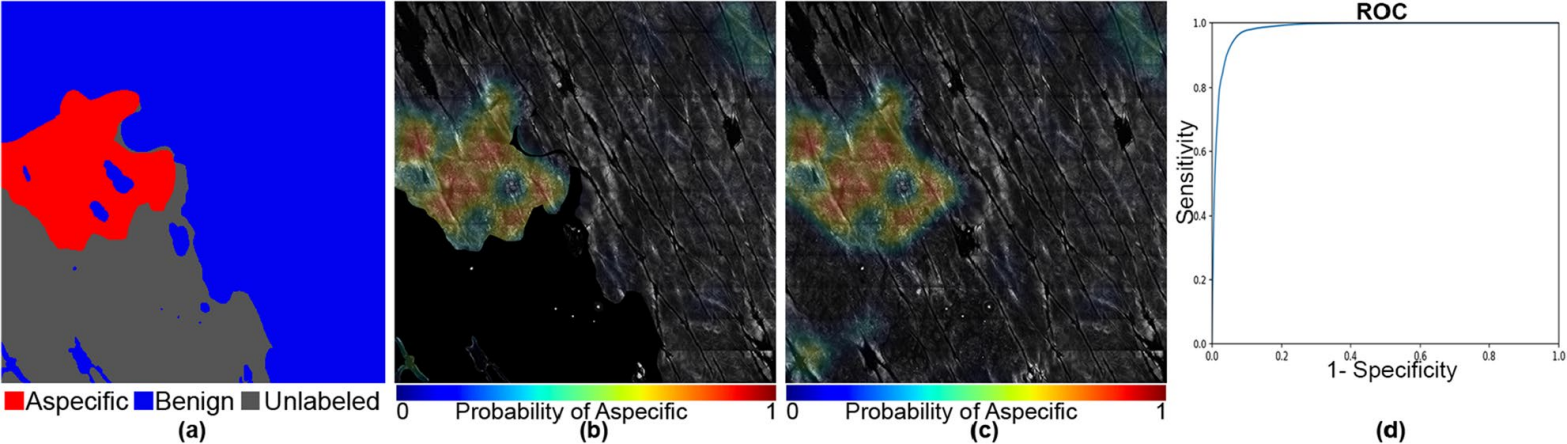
An example of both aspecific, unlabeled, and benign patterns with an AUC of 0.982 and a Dice coefficient of 0.83. The mask and the heat map images are generated using OpenCV 4.5.1.48 (https://pypi.org/project/opencv-python/) library in Python 3.7.4 (https://www.python.org/downloads/release/python-374/). The images and the legends are put together using Photoshop CC 2018.

**Figure 6:**
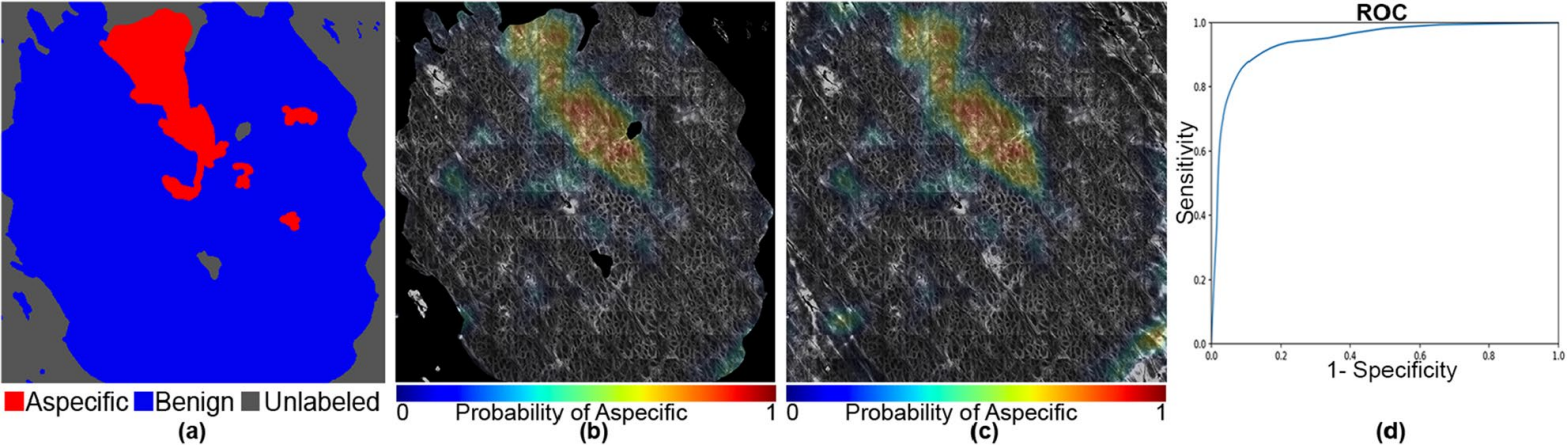
An example of aspecific, unlabeled, and benign patterns with an AUC of 0.942 and a Dice coefficient of 0.67. The mask and the heat map images are generated using OpenCV 4.5.1.48 (https://pypi.org/project/opencv-python/) and the ROC curve is generated using matplotlib 3.3.3 libraries (https://matplotlib.org/users/installing.html) in Python 3.7.4 (https://www.python.org/downloads/release/python-374/). The images and the plot, together with the legends is put together using Photoshop CC 2018 (https://prodesigntools.com/adobe-cc-2018-direct-download-links.html).

**Figure 7:**
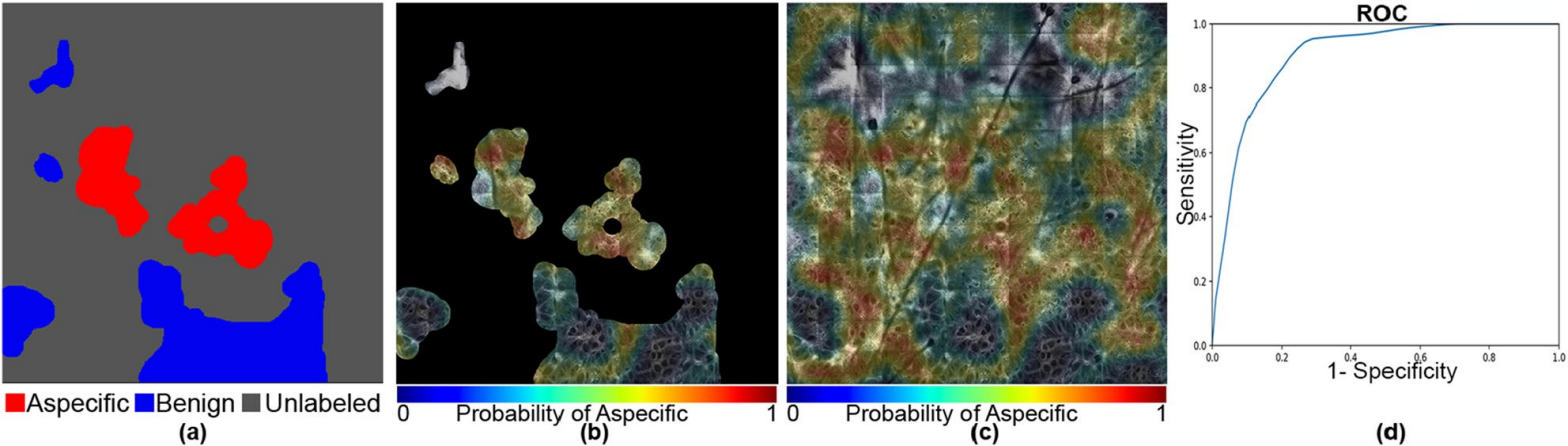
An example featuring aspecific, unlabeled, and benign patterns with an AUC of 0.903 and a Dice coefficient of 0.79. The mask and the heat map images are generated using OpenCV 4.5.1.48 (https://pypi.org/project/opencv-python/) library and the ROC curve is generated using matplotlib 3.3.3 libraries (https://matplotlib.org/users/installing.html) in Python 3.7.4 (https://www.python.org/downloads/release/python-374/). The images and the plot, together with the legends are put together using Photoshop CC 2018 (https://prodesigntools.com/adobe-cc-2018-direct-download-links.html).

**Figure 8:**
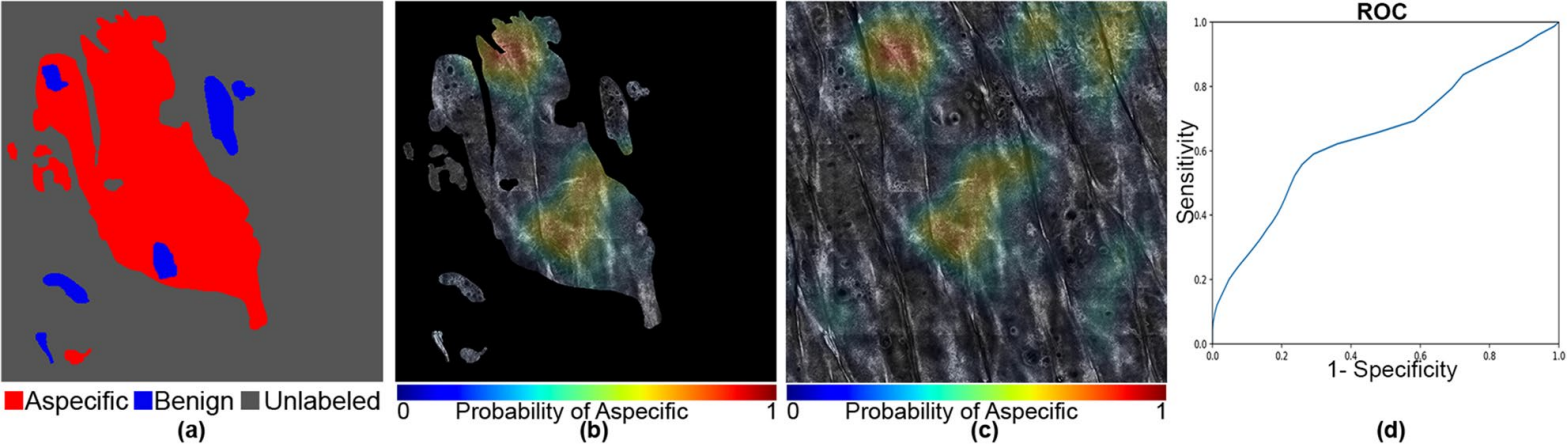
An example with a majority unlabeled or aspecific patterns with an AUC of 0.646 and a Dice coefficient of 0.45. The mask and the heat map images are generated using OpenCV 4.5.1.48 (https://pypi.org/project/opencv-python/) and the ROC curve is generated using matplotlib 3.3.3 libraries (https://matplotlib.org/users/installing.html) in Python 3.7.4 (https://www.python.org/downloads/release/python-374/). The images and the plot, together with the legends are put together using Photoshop CC 2018 (https://prodesigntools.com/adobe-cc-2018-direct-download-links.html).

**Figure 9:**
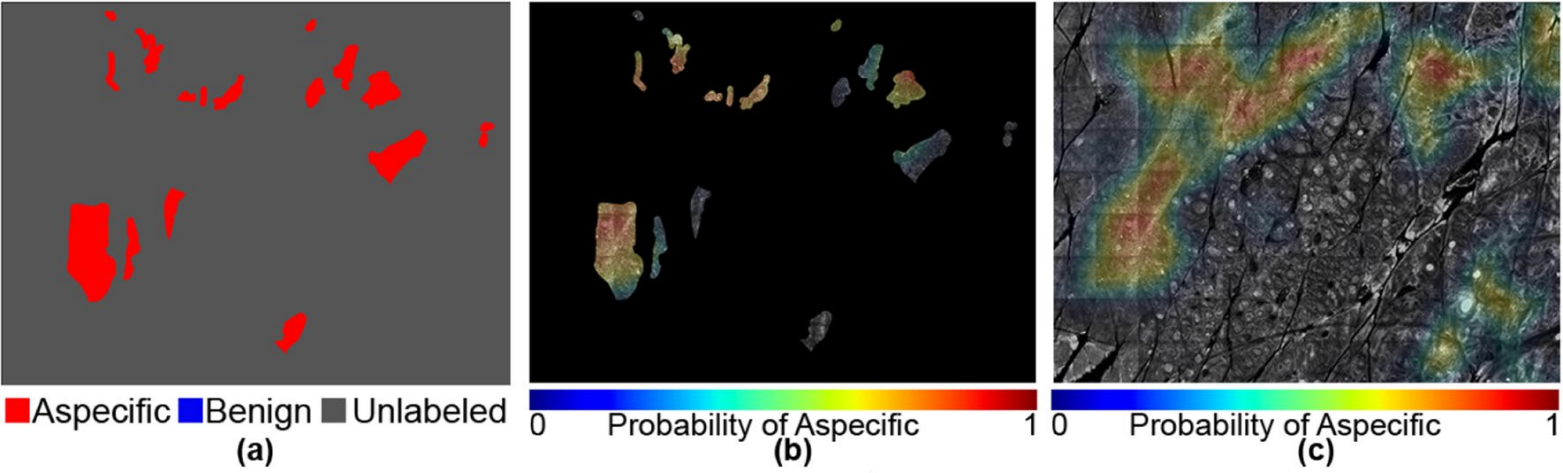
An example with no benign patterns and a Dice coefficient of 0.75. The mask and the heat map images are generated using OpenCV 4.5.1.48 (https://pypi.org/project/opencv-python/) library in Python 3.7.4 (https://www.python.org/downloads/release/python-374/). The images and the legends are put together using Photoshop CC 2018 (https://prodesigntools.com/adobe-cc-2018-direct-download-links.html).

**Figure 10:**
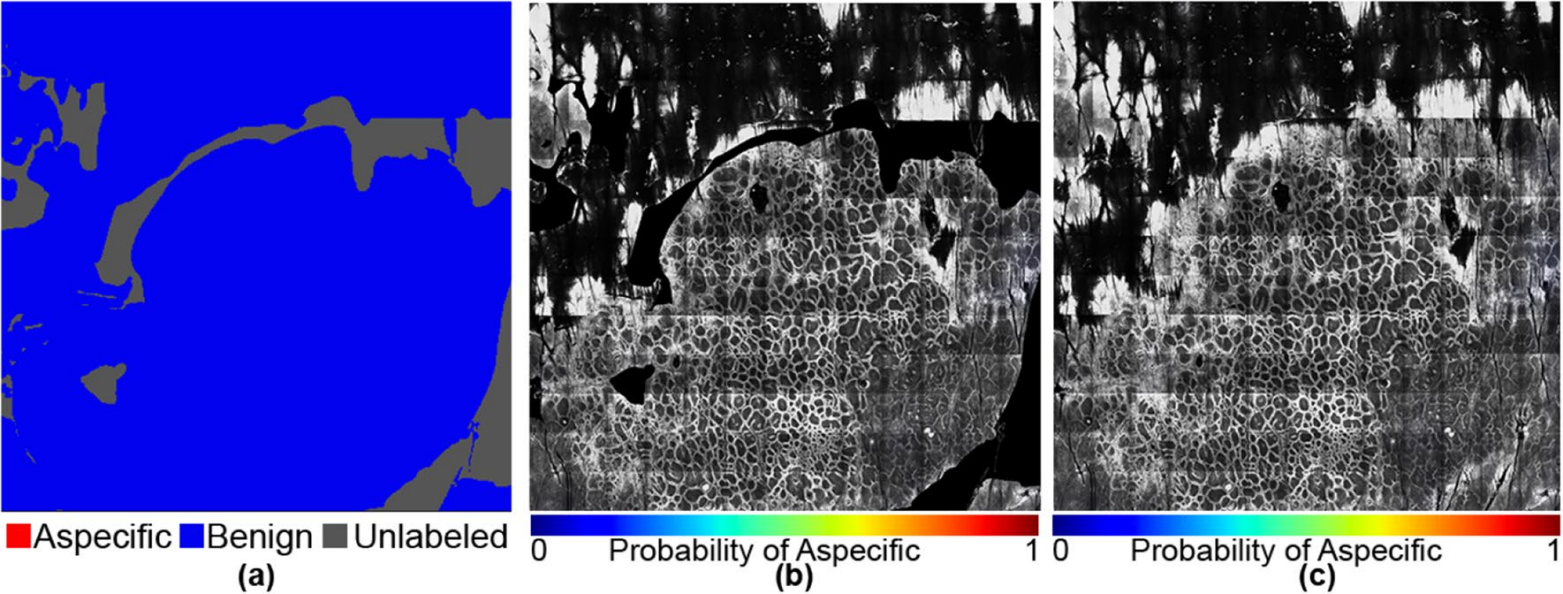
An example with no aspecific patterns. The mask and the heat map images are generated using OpenCV 4.5.1.48 (https://pypi.org/project/opencv-python/) in Python 3.7.4 (https://www.python.org/downloads/release/python-374/). The images and the legends are put together using Photoshop CC 2018 (https://prodesigntools.com/adobe-cc-2018-direct-download-links.html).

In Figs. 5, 6, 7, 8, 9 and 10, we show exemplary results for six different mosaics. In each figure, panel (a) shows the ground truth labeling of the experts, panel (b) the CAM map generated by the model with unlabelled areas masked out, and panel (c) the CAM including the unlabelled areas. Finally, panel (d) shows the corresponding ROC curves and AUC metrics for the first four cases. In the last two mosaics, only one of the classes is present in the ground truth, so the ROC curve and AUC metrics cannot be calculated, as those metrics depend on the accuracy of the method at distinguishing between two classes compared to their relative frequency in the ground truth. We note though that in the all benign case (Fig. 10) the algorithm did not generate any false positives.

**Figure 11:**
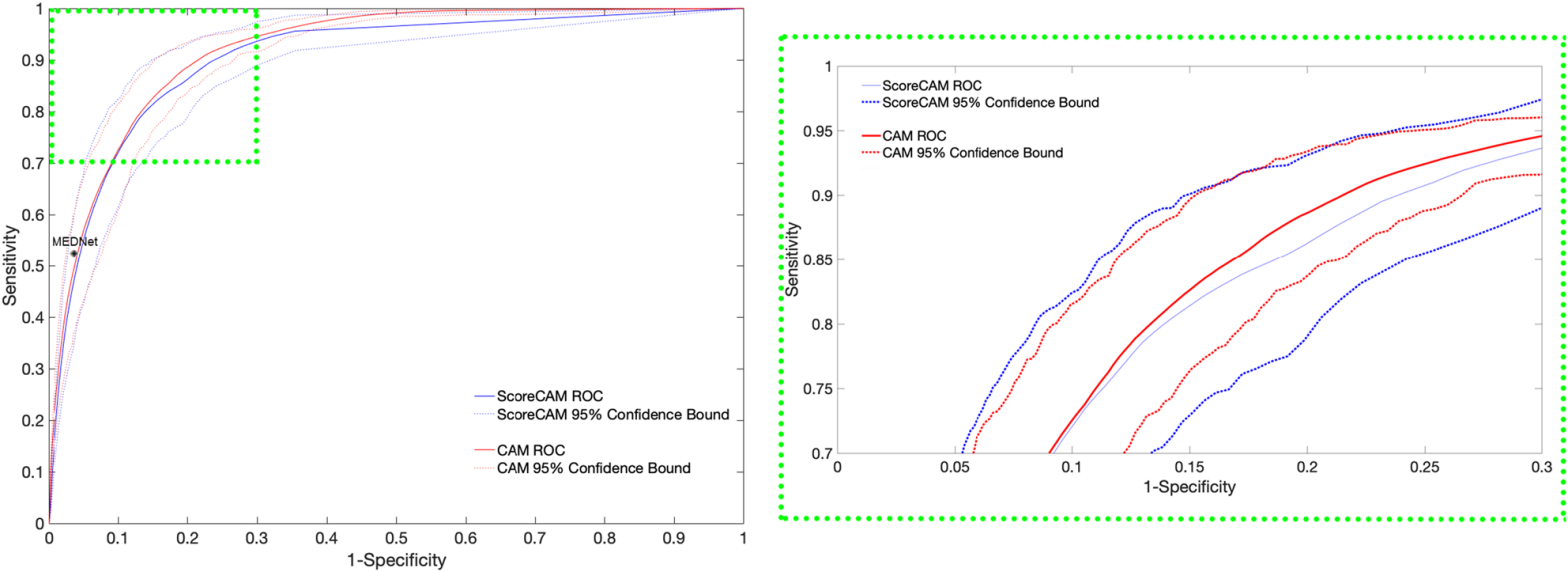
Comparison between the ROC of CAM and the ScoreCAM approaches. Solid line shows average ROC line of 1000 bootstrap runs and the dashed line shows the $95\%$ confidence interval for the bootstrap experiments. For comparison, we also show the performance of the fully supervised approach, MED-Net^3^ as a black dot in the graph in the left panel.

We also present a comparison between the best performing CAM and ScoreCam models in Fig. 11. We performed 1000 bootstrapping experiments, where we randomly selected with replacement 31 mosaics from the test set and calculated the ROC for each realization. The average ROC (solid lines) and $95\%$ confidence interval (dashed lines) obtained for these experiments is presented in Fig. 11. The green inset in the figure shows a blown-up version of the upper left corner of the graph, the high sensitivity and specificity region.

**Table 2:** The AUC and Dice scores calculated for each run as a function of step size and the width of the Gaussian weighting.

CAM generation	CAM		ScoreCAM	
Run	AUC	Dice	AUC	Dice
Step size: 25, (no Gaussian)	0.968	0.770	0.927	0.603
Step size: 25, 1	0.968	0.771	0.928	0.609
Step size: 25, 2.5	0.969	0.775	0.933	0.623
Step size: 25, 5	0.969	0.778	0.933	0.628
Step size: 25, 6	0.969	0.778	0.932	0.627
Step size: 25, 7	0.969	0.777	0.931	0.624
Step size: 25, 8	0.969	0.776	0.930	0.621
Step size: 25, 9	0.965	0.775	0.926	0.622
Step size: 25, 10	0.962	0.773	0.923	0.618
Step size: 50, (no Gaussian)	0.961	0.768	0.921	0.603
Step size: 50, 1	0.962	0.768	0.922	0.607
Step size: 50, 2.5	0.962	0.772	0.926	0.620
Step size: 50, 5	0.962	0.773	0.926	0.623
Step size: 50, 6	0.962	0.772	0.932	0.627
Step size: 50, 7	0.962	0.771	0.931	0.624
Step size: 50, 8	0.962	0.770	0.930	0.622
Step size: 50, 9	0.958	0.768	0.926	0.620
Step size: 50, 10	0.955	0.766	0.923	0.617
Step size: 100, (no Gaussian)	0.953	0.761	0.910	0.591
Step size: 100, 1	0.954	0.762	0.912	0.597
Step size: 100, 2.5	0.955	0.767	0.916	0.610
Step size: 100, 5	0.954	0.763	0.913	0.600
Step size: 100, 6	0.954	0.760	0.910	0.595
Step size: 100, 7	0.953	0.758	0.908	0.592
Step size: 100, 8	0.953	0.756	0.906	0.589
Step size: 100, 9	0.950	0.754	0.902	0.588
Step size: 100, 10	0.946	0.752	0.899	0.588

Step size is given in pixels.

## Materials and methods

### Ethical guidelines

All mosaics were collected under the required IRB (USA) and Ethics Committee (EU) approvals with written informed consent obtained from the subjects. All the data is de-identified (patient metadata was removed). All experiments were performed in accordance with relevant guidelines and regulations.

### Problem formulation

As discussed in “Introduction”, the goal of this study is to perform weakly supervised semantic segmentation on RCM images, meaning the network takes an image and an image-level label as an input and outputs pixel-wise labels for the image. We accomplish this by first training a classification network and then modifying the final layers to create CAMs. Convolutional neural networks (CNN) based classification models can be easily modified to create rough pixel-level annotations by using the output of the final convolutional layer. This final layer is traditionally recognized as the output of the feature extraction portion of the segmentation network and it contains the discriminative sections of the image used to determine the final classification decision.

Due to the difficult nature and small size of our data set, we wanted a lightweight network with a small number of parameters, which would have less demanding training requirements compared to alternative networks of comparable accuracy. We selected Efficientnet^12^ due to its proven ability to accomplish classification tasks with high accuracy while using a relatively small number of parameters ($\sim 7$ million). Efficientnet was then modified in two ways to generate the CAMs—(1) using the original method described by Zhou et al.^13^ of extracting information from the feature extraction layer (which we refer to as the traditional CAM method) and (2) using an alternative approach denoted ScoreCAM^14^, which was introduced as an attempt to overcome certain shortcomings of the traditional CAM method, as discussed in “ScoreCAM generation”.

### Efficientnet overview

The main consideration in the design of Efficientnet models^12^ is to scale the layers of a convolutional neural network uniformly with a constant ratio to increase the accuracy of classification without changing the functions of the hidden layers or exceeding the system’s capabilities in terms of memory and computational burden. Several versions of Efficientnet with different sizes and depths are available. Each version provides a different topology offering different performance for a given training budget, defined by the size of the data and the computational resources available during training^12^. The details of the model can be found at^12^.

### Traditional class activation map generation

**Figure 12:**
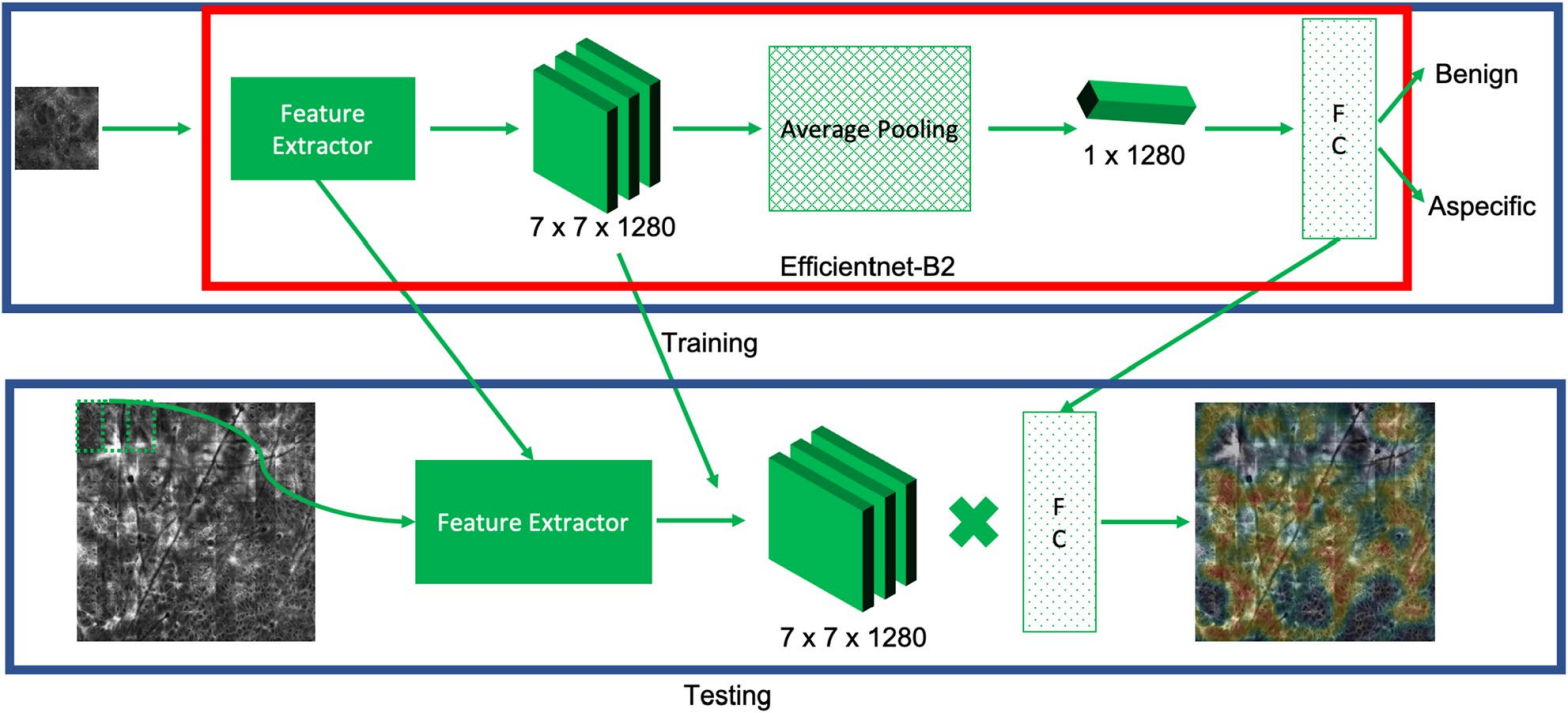
An overview of the algorithm—Efficientnet-B2 is trained for classification in the standard manner (note that FC denotes the fully connected final layer). In testing, the mosaic is broken up into patches that are fed to the feature extractor. The extracted features are then multiplied by the weights in the fully connected layer to create a CAM.

We modified the Efficientnet classification model to generate CAMs. The Efficientnet classification topology can be divided into two main parts as shown in Fig. 12. The convolutional layers in the initial part of the network compose the feature extraction portion, which takes an image as an input, processes it using multiple cascaded convolutional layers, and outputs a multi-channel feature representation with a spatial size typically smaller than the input. The feature extraction part of the network is followed by fully connected classification layers, which classify the feature representation of the images into one of the known classes. These two parts of the network are connected to each other via a global average pooling (GAP) layer. The GAP layer reduces the feature representation of each image into a single vector, by averaging the values in each feature channel. The final classification probability of each class is calculated by calculating the weighted sum of the entries of this feature vector representation. The weights in the summation are the learnable parameters of the final fully connected layer.

A schematic of the WSL method is presented in Fig. 12. In order to generate a CAM for a given class, we bypass the GAP layer and directly calculate the weighted sum of the output of the feature extraction layer, by taking the Hadamard product of the respective fully connected layer weight with the feature representation. All values are then normalized to between 0 and 255, and the output is upsampled using bilinear interpolation to the original image size.

### ScoreCAM generation

ScoreCAM^14^ is a recent CAM generation method that aims to overcome the shortcomings of the regular CAM method caused by its dependence solely on the feature maps generated during training. In addition, reliance on the global pooling layer can cause false confidence in parts of the image, causing less important sections to be weighted at least as high or higher than the most important sections^14^. In ScoreCAM the importance of activation maps are encoded by the global contribution of the corresponding input features instead of the local sensitivity measurement^14^. This is accomplished in two phases. In the first phase, the input images are passed through the neural network model to obtain the output classification class label and activations obtained at a level pre-selected by the user. These activations are then normalized using a softmax operation and upsampled to the size of the input image. In the second phase, initially, the activations are multiplied (Hadamard multiplication) with the input image to get a masked input $\textit{M}^k$ as

$$\begin{aligned} M^{k} = A^{k} * I \end{aligned}$$

where $A^k$ is the weights of the *k*’th layer and *I* is the input image. Then, the final scores $S_{k}$ are calculated by passing the masked input through the network followed by a softmax output. These scores represent the importance of each respective activation map. A single activation map is formed by a weighted sum of all the activations for the target class as in the regular CAM method.

$$\begin{aligned} A^{tot} = \sum _{k=1}^{K} S_{k} A^{k} \end{aligned}$$

Finally, a pixel-wise ReLU is applied to $A^{tot}$ to filter out all negative values of $A^{tot}$, which represent those features that did not have a positive influence on the class of interest.

### Model training

The Efficientnet-B2 model used in our study contained $\sim 7$ million learnable parameters. We trained the model for 500 epochs (without early stopping) with an initial learning rate of $1 \times 10^{-3}$, which decreased by 0.9 every 10 epochs, using stochastic gradient descent with a momentum of 0.9 and a weight decay of $5 \times 10^{-4}$ and a dropout rate of 0.75 to avoid overfitting. We used a batch size of 128 images. The data augmentation performed included random cropping to $256 \times 256$ pixels, random horizontal flip, random vertical flip, and grayscale jitter for brightness and contrast, noise, and blurring. So the final input to the network is a batch of patches of size 256-by-256 pixels. We used binary cross-entropy with a logit loss function. For our purposes, the target value is a binary classification problem—either the image contains the aspecific label or it does not. After the training, the class activation maps of the classification model were used to infer the segmentation labels for the aspecific class.

### Inference of segmentation labels in mosaics

The final model is capable of conducting classification at the level of patches of size $256 \times 256$ pixels which corresponds to $\sim 0.5\,{\hbox {mm}}$-by-0.5 mm area. However, since as described above our ultimate aim is to infer pixel-wise labels for the large field of view mosaics typically used in the clinical setting for diagnostic analysis and since these mosaics are composed of several RCM images collected from multiple, neighboring fields of views in a scanning (e.g. raster) fashion and concatenated (or stitched) together, we took the following steps.

#### Sliding window processing

In order to obtain segmentation labels over the full mosaic area, the mosaic was processed in an overlapping sliding window fashion with a window size of 256-by-256 pixels. The partitioning of patches from the entire mosaic can cause the network to lose contextual information, so processing the mosaic in an overlapping window fashion resulted in evaluations of the same pixel at different spatial locations in different patches as well as to help mitigate false segmentations. Step sizes of 25, 50, and 100 pixels were investigated.

After creating a patch, it was classified as either aspecific or benign, and the corresponding CAM was generated as described in “Traditional class activation map generation”. We wanted to generate an entire mosaic, and simply stitching these values together was not logical—combining a CAM for an aspecific patch with a CAM for a benign patch would not make sense. Instead, we wanted to boost the CAMs for aspecific patches while suppressing those for benign patches to clearly highlight the areas of interest. We accomplished this by multiplying the probability that the patch is “aspecific class” by the normalized CAM values of each patch. After processing a window, the results at the overlapping section were averaged together.

#### Gaussian weighting

However, we note that due to boundary conditions in the convolutional layers, the model is typically more confident at identifying the regions at the center of the patches, and averaging the patches at overlap regions does not take this effect into account. Therefore, in order to compensate, when aggregating in a sliding window fashion, we gave more weight to the decision values at the center of the patch compared to those at the edges. This was accomplished by implementing a two dimensional Gaussian kernel, defined as

$$\begin{aligned} g(x,y) = \frac{1}{\sqrt{2\pi \sigma ^{2}_{1}}} \exp {\left( -\frac{1}{2}\frac{x - \mu _{1}}{\sigma ^{2}_{1}} \right) } \cdot \frac{1}{\sqrt{2\pi \sigma ^{2}_{2}}} \exp {\left( -\frac{1}{2}\frac{y - \mu _{2}}{\sigma ^{2}_{2}} \right) } \end{aligned}$$

where $\mu$ is the mean ($\mu _{1} = \mu _{2} = 0$), $\sigma _i$, $i = 1,2$ are user-defined parameters representing the variance of the distribution and *(x,y)* are the input pixel values. Each CAM generated by the network is weighted by this Gaussian kernel that suppresses the values of the pixels that are further away from the center of the patch. The amount of suppression is defined by the variance of the Gaussian kernel, $\sigma _i$. We tested the method with several $\sigma$ values and report our findings in “Results”.

## Discussion

Evaluating the patch-level classification accuracy of Efficientnet assured us that our implementation was trained successfully and allowed us to proceed with CAM generation and mosaicking. As shown in Table 2, the values of the parameters (overlap and $\sigma$) over the range we tested had a minimal effect on the AUC results, with a difference of less than 5% between the lowest and highest AUC. As also seen in Table 2, the highest performing ScoreCAM trial was equal to the second-highest performing traditional CAM generation trial. Moreover, the range of AUCs for ScoreCAM (4%) was slightly larger than those seen in the regular CAM generation method (3%). However, the choice of CAM generation method had a larger impact on the Dice scores, with the optimal threshold being 0.5 for traditional CAM generation and 0.4 for ScoreCAM. Traditional CAM generation performed significantly better than ScoreCAM, with maximum scores of 0.778 and 0.627, respectively. The ScoreCAM method was designed to remove noise, but in our application it seems that it may be suppressing values in the process, leading to smaller values that do not pass the Dice threshold. Again, step size and $\sigma$ did not have a significant impact on results, with a range of less than 1% in both CAM generation methods.

Comparing against state of the art in the literature^3^, the performance of the fully supervised approach, MED-Net^3^ falls into the $95\%$ confidence interval of the weakly supervised approach as shown in Fig. 11. We used the results of the stratified cross-validation experiments reported in Kose et al.^3^, which is the same setting that we used in our experiments here. However, in the interests of a fair comparison, we point out that MED-Net solved a multiclass segmentation problem (six classes), which is a significantly harder task. Therefore, MED-Net might be expected to outperform the weakly supervised approach described here if MED-Net were trained for only two class segmentation, similar to the task carried out here. On the other hand, of course, MED-Net takes advantage of far more highly resolved (pixel-level) expert labeling in training. This comparison suggests that the results reported here are promising but that more work must be done to bring our method to the same level of accuracy as the fully-supervised state of the art^3^.

Further examination of the weakly supervised segmentation results for individual mosaics in Figs. 5, 6, 7, 8, 9 and 10 reveals how ROC curves and AUC values vary across mosaics. In Figs. 5 and 10, we achieved nearly perfect segmentation results with only a small section at the upper right corner of Fig. 5 incorrectly classified as aspecific. However, we note that this section had a lower activation value than the correctly identified aspecific section located in the middle left of the mosaic, indicating the network was less confident that the area was aspecific.

However, in a few cases, as shown in Fig. 7, activations for the aspecific region occurred in the unlabeled portions of the mosaic. In our quantitative analysis, we are limited to the areas that were labeled by our expert readers and are therefore unable to evaluate the accuracy of the network on these unlabeled areas. In other areas, as illustrated in Fig. 8, our model did not perform satisfactorily as it missed parts of the aspecific pattern, resulting in a very low AUC (0.646). We speculate that the model requires more data to be able to correctly generalize across the large spectrum of variation in aspecific patterns in RCM. We expect, therefore, that in the future, with the addition of more data in our training process (which will itself be greatly facilitated with this weakly-supervised approach as the burden on labelers is reduced so dramatically) we will be able to include more variability in the aspecific class training and thereby reduce false negatives.

Our results suggest that our CAM-based approach may increase diagnostic efficiency as it enables quick and precise identification of mosaics containing the aspecific pattern. This approach may also save lives by ensuring that all potential melanomas are identified and flagged for biopsy or further expert review. In the future, this CAM based approach could also be incorporated into a melanoma risk score to increase diagnostic confidence and/or accuracy (in the case of a non-expert). However, as both the precise location and extent of atypia factor into melanoma diagnosis, segmentation performance of the algorithm would benefit from more extensive studies using both a larger number of lesions and labeling from several experts.

To bring this work to the clinical setting, clinicians must continue to be systematically consulted on the results. Their feedback on metrics used to evaluate the mosaics, particularly the threshold at which we should classify a patch as aspecific, would be particularly valuable. As discussed in “Patch-level classification performance”, the model cannot accurately identify all instances of aspecificity when using a softmax threshold of 0.5, so a panel of experts could also provide useful feedback on how conservative this threshold should be. Exploring in detail the incorrect classifications and the classifications of the unlabeled regions would help us to better interpret how the network is performing. Perhaps most importantly, clinicians should guide the process of determining, which results are the most important, and also how to best visualize and interpret results in a clinical setting. We plan to carry out an extended feasibility study, with the logical next step of expanding the capabilities of the network to only use mosaic-wise labels for training. One possible solution to this challenging problem would be to adopt a method similar to the one presented in^15^, in which we would combine multiple instance learning and CAMs to achieve segmentation of diagnostically important portions of RCM images in a substantially larger dataset. Another future step in our research will be to attempt to train a similar model to the one presented here but expand segmentation to all six architectural patterns in an effort to achieve a specific diagnosis (e.g. congenital nevus versus dysplastic nevus versus melanoma) rather than a binary benign versus malignant category.

## Conclusion

In previous reports, semantic segmentation of RCM images was successfully carried out using fully supervised segmentation models such as MED-Net. However, due to excessive labeling requirements, those models are not feasible to apply in many practical settings, nor to rapidly expand to different RCM image analysis problems. In this work, we examined the feasibility of performing weakly supervised semantic segmentation on RCM images using class activation maps. The method presented here offers the potential of an interpretable diagnostic aid to clinicians by segmenting diagnostically significant/suspicious spatial regions in an image mosaic while providing classification labels with an AUC of 0.969. However, the results we present fell within the 95% confidence interval of MED-Net’s performance, indicating that a weakly supervised model, when extended to six patterns, might also provide the kind of diagnostic reasoning that is much desired by the clinical community while using an automated algorithm.
